# Preventing Internalizing Problems in 6–8 Year Old Children: A Universal School-Based Program

**DOI:** 10.3389/fpsyg.2016.01928

**Published:** 2016-12-15

**Authors:** Eugenie Pophillat, Rosanna M. Rooney, Monique Nesa, Melissa C. Davis, Natalie Baughman, Sharinaz Hassan, Robert T. Kane

**Affiliations:** School of Psychology and Speech Pathology, Curtin UniversityPerth, WA, Australia

**Keywords:** anxiety, prevention, lower primary school children

## Abstract

The Aussie Optimism Program: Feelings and Friends (AOP-FF) is a 10 week, universal mental health promotion program based on social/emotional and cognitive and behavioral strategies. The aim of the current study was to evaluate the efficacy of a universal Cognitive Behavioral Therapy based program in preventing and reducing internalizing problems in 6–8 year olds (Years 1–3 in Australia). Year 1–3 students from a low SES primary school (*N* = 206) were randomly assigned in classes to either an intervention or a control group and assessed at baseline and post-test. Results showed a significant (*p* = 0.009) and small to moderate (partial eta-squared = 0.034) pre-post decrease in parent-reported anxiety symptoms for the intervention group, in conjunction with a non-significant (*p* = 0.708) and negligible (partial eta-squared = 0.001) pre-post increase for the control group. A larger randomized controlled trial assessing longer term effects is needed. In addition the program needs to be simplified for year 1–2 students with a separate more developmentally appropriate program for year 3 students.

## Introduction

### Prevention of internalizing disorders in early childhood

The two internalizing disorders, anxiety and depression, are recognized as the most common and increasingly prevalent childhood disorders (Tonmyr et al., 2011; Essau et al., 2012a). Anxiety and depression have been posited to be temporally linked (Hansell et al., 2012), and some researchers have argued that the high comorbidity and symptoms overlap indicate that the two internalizing disorders are part of a single entity or personality construct (Watson et al., 2005). Anxiety and depression are evident in early childhood and are often associated with significant daily impairment and interference with a child's interpersonal and academic functioning (Roberts and Bishop, 2003; Hirshfeld-Becker et al., 2010; Trudeau et al., 2012). While clinical trials of anxiety and depression intervention programs indicate that internalizing disorders in late childhood and early adolescence may be effectively treated (Monshouwer et al., 2012), a significant proportion of children continue to experience difficulties post-intervention (Pössel et al., 2011). As a consequence, it is critical that the emphasis is on prevention in dealing with internalizing disorders in children, and for prevention to begin sooner rather than later.

The transition to primary school has been identified as a vulnerable stage for many children (Tomb and Hunter, 2004; Goodwin et al., 2012) due to several factors such as spending a significant period away from their families for the first time; entering a new social environment where the need for social approval and to fit in with peers becomes significant; and being evaluated in terms of how well they perform compared to their peers (McLoone et al., 2006; Goodwin et al., 2012). As such, manifestations of social, separation and performance anxiety may emerge when children enter primary school.

### Risk and protective factors for internalizing disorders in childhood

Prevention programs can work by either reducing the influence of *risk* factors, or by enhancing and developing *protective* factors in order to promote resiliency in children (Dadds et al., 2000; Romeo et al., 2011). As with risk factors, protective factors have generally pertained to individual child characteristics (e.g., temperament, cognitive style, social skills, self-efficacy, and esteem) or environmental and learning experiences such as effective parenting, high family cohesion, positive parent-child relationships (Davis et al., 2000; Dick-Niederhauser and Silverman, 2004; Jakobsen et al., 2012). The presence of *positive social support networks* have been found to be an important protective factor against the development of emotional disorders, particularly in the face of stressful life events (Heaney et al., 2010; Grav et al., 2012; Tsai et al., 2012). Another important protective factor is a child's *coping skills repertoire* (Wright et al., 2010), with problem focused coping strategies being associated with greater mastery and positive psychological adjustment than avoidant or emotion focused coping skills (Dick-Niederhauser and Silverman, 2004; Klein et al., 2011). The role of an avoidant coping style in the maintenance of internalizing disorders, particularly anxiety, is widely recognized (Barlow, 2002; Eisenberg et al., 2012). For instance, in the avoidance learning model, it is hypothesized that fears or phobias will fail to extinguish if one learns to avoid the feared stimulus (Dymond et al., 2011). McLoone et al. (2006) argued that to effectively treat children with comorbid anxiety and depression, components of treatment should include enactive programming (e.g., proactive engagement in therapy via exposure and pleasant activity scheduling).

It has been recommended that building up social and emotional competencies should begin in preschool (Greenberg et al., 2003; Conner et al., 2011). Emotional competence includes a child's capacity to recognize, monitor and regulate their emotions, appreciate the perspectives of others, establish prosocial relationships and goals, as well as effective interpersonal skills and problem solving abilities (Hessler and Katz, 2010). Emotional awareness and knowledge in young children have been found to be predictive of social competence and emotional well-being in later childhood (Fine et al., 2003; Oades-Sese et al., 2011). Moreover, emotional competence has been linked with positive school and classroom adjustment, enhanced academic learning, and found to be predictive of psychological health in later childhood (Linaries et al., 2005; Miller et al., 2006).

Social competence is a significant prerequisite in the formation of positive, stable, and enduring peer relationships (Gagnon and Nagle, 2004). Furthermore, peer relationships have been implicated as mediating and protective factors in psychological adjustment, as well as encouraging more positive school transitions, better school adjustment, increased achievement and school liking, decreased school avoidance, protection against victimization in school, and more healthy adjustments to negative life experiences (Bukowski and Adams, 2005; Masten, 2005; Kochel et al., 2012).

### Preventive interventions in the field

Prevention programs can be either universal, where the program is implemented for a designated population regardless of risk, selected, where the program is targeted to a population at risk, or indicated, where the program is aimed at individuals displaying early symptoms of the disorder (Dick-Niederhauser and Silverman, 2004). Advantages of universal programs include their ability to avoid the stigma of labeling that is inherent in selective and indicated interventions, avoiding the potential to overlook “at risk” children due to inadequate screening measures, as well as the ability to provide the opportunity for all children to reap the benefits of the program and develop resiliency (Donovan and Spence, 2000; Kösters et al., 2012).

The majority of prevention programs for internalizing disorders have targeted older children (e.g., middle or later primary years; Barrett and Turner, 2001; Lowry-Webster et al., 2001; Quayle et al., 2001; Roberts et al., 2003, 2004; Rooney et al., 2006, 2013a,b; Fox et al., 2012; Johnstone et al., 2014; Myles-Pallister et al., 2014). There are comparatively few prevention programs specifically targeting internalizing disorders in younger children. One such program is the *Queensland Early Intervention and Prevention of Anxiety Project* (QEIPAP; Dadds et al., 1997) which is adapted from the Coping Koala prevention program (Barrett et al., 1994) and employs primarily, cognitive-behavioral approaches. Dadds and his colleagues evaluated the efficacy of the program in a sample of 7–14 year olds, who were randomly assigned to a control or 10-week school based child and parent focused psychosocial intervention group. While both groups demonstrated improvements in anxiety symptomology post-intervention, these gains were maintained at 6 month and 2 year follow-up only in the intervention group (Dadds et al., 1997, 1999).

The PATHS (Promoting Alternative Thinking Strategies) curriculum is a widely known program designed to promote social and emotional competence through cognitive skills building in Grades 1–3 children (Kam et al., 2004; Saltali and Deniz, 2010). In evaluating PATHS, it was found that compared to controls, intervention children showed improvements on social problem-solving, emotional understanding, and teacher ratings of internalizing and externalizing problems (Kam et al., 2004; Saltali and Deniz, 2010). However, PATHS doesn't target internalizing problems and consequently the effect sizes found for internalizing problems have been 0.22 in the low range. PATHS is also designed as a multiyear program, having the additional drawback of being potentially cost ineffective due to the resources needed to run the program over a lengthy period of time.

Supporting the findings of PATHS, evaluations of a similar program, the *I Can Problem Solve* program (Anliak and Sahin, 2010) was found to significantly improve cognitive problem solving abilities, and reduce impulsivity and inhibition, with enduring effects at 1 year follow up (Shure, 1997; Anliak and Sahin, 2010). Support was also found for the *Incredible Years Classroom Social Skills and Problem Solving Curriculum* (*Child Dinosaur Program;* Webster-Stratton and Reid, 2004), which also looks at strengthening social and emotional competence in young children. However, all effects for internalizing problems have been in the low to moderate effect size range (Durlak et al., 2011).

While existing programs designed for younger children have targeted social and emotional competence, a program specifically targeting the internalizing disorders anxiety and depression does not presently exist and effect sizes of existing established programs on internalizing problems have been low to moderate (Durlak et al., 2011). A comprehensive and cost effective program, targeting internalizing problems, to be implemented widely throughout the school system, needs to be developed and evaluated. Designed to meet the developmental needs of 6–8 year old children, this program should combine evidence-based cognitive behavioral strategies, while simultaneously having a strong focus on building social and emotional competence.

### Rationale for the current study

The aim of the current study was to evaluate the efficacy of a universal Cognitive Behavioral Therapy based program in preventing and reducing internalizing problems in 6–8 year olds (Years 1–3 in Australia).

#### Areas to be targeted

Based on the evidence reviewed, a focus of the program was to promote children's emotional knowledge, awareness, and emotional regulation. A further component included was improving prosocial friendship skills and effective social problem-solving skills, as well as to encourage perspective-taking. These skills are designed to minimize interpersonal conflict, decrease rejection and negative appraisals from peers, and enhance status among peers, thereby increasing children's *positive support network* (Spence, 2003; Glick and Rose, 2011). In addition, the role of *effective coping skills* is important in promoting resiliency, self-efficacy and a sense of mastery. Rodebaugh et al. (2004) have argued that to address depressive and anxiety disorders from a cognitive-behavioral theoretical perspective, behavioral strategies such as enactive programming (e.g., assisted graded exposure, providing strategies to face the feared stimulus) should be included. The inclusion of enactive programming maximizes chances of addressing both types of internalizing disorders. As such, the emphasis of the program is also to build upon existing programs by providing children with a repertoire of effective coping skills that are directly linked to anxiety and depression. These include relaxation training, pleasant activity scheduling, facing one's fear instead of avoidance, and seeking social support when needed. No study to date has included behavioral strategies in combination with building social and emotional competence. In this way, the current program targeted internalizing disorders more specifically than any other program to date.

#### Universal school-based approach

The current program employed a universal approach to prevention, and was available to all children in the school rather than limited to those “at risk.” As universal programs are offered to all children, it is logical for the program to be part of the school curriculum. Previous researchers have found that preventive programs that are incorporated into the existing school health curriculum increase their cost-effectiveness, utility, and accessibility (Calear and Christensen, 2010; Kösters et al., 2012).

In sum, the current program adhered to several important factors salient to preventive programs. It:

1. Encompassed a developmental framework,
2. Integrated a variety of successful, evidence-based approaches,
3. Incorporated existing risk and resiliency literature, with a dual focus on social and emotional competence, and on developing an effective repertoire of coping skills salient to internalizing difficulties,
4. Employed a universal approach, utilizing the school environment as a place where change can occur.

#### Hypotheses

It is hypothesized that:

H1a *Universal Prevention Effects*: At post-test, children in the intervention group will have lower depression and anxiety scores on self and parent reports, improved social skills and more well-developed emotion knowledge than children in the control group.
H1b *Universal Prevention Effects*: At post-test, children in the intervention group who score in the normal range at pre-test will maintain a healthy status (i.e., parental and self-report measures of internalizing symptoms within the normal range).
H2 *Selected or Indicated Prevention Effects*: Children in the intervention group who were initially identified as being “at risk” (i.e., who exhibit symptoms of internalizing difficulties at pre-test) will exhibit a significant decline in internalizing difficulties at post-test.

## Methods

### Ethics statement

Informed consent was obtained from both next of kin and the children in written form. The child consent form contains information that the child can understand and has their signature of consent on it and the case is the same for the parents. This study has been approved by the Curtin University Human Research Ethics Committee. If needed, verification of approval can be obtained either by writing to the Curtin University Human Research Ethics Committee, c/- Office of Research and Development, Curtin University of Technology, GPO Box U1987, Perth, 6845 or by telephoning 9266 2784.

### Participants

**Figure 1:**
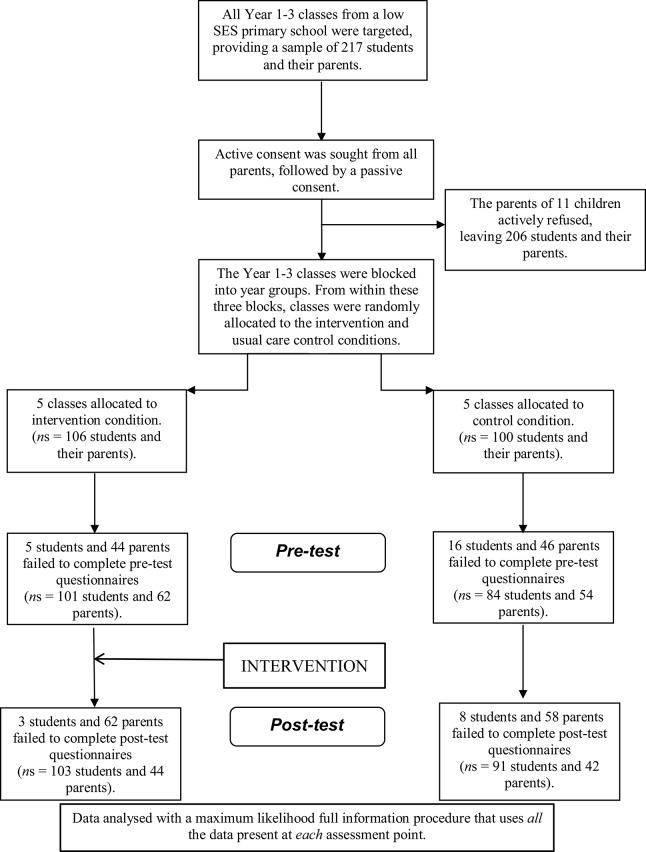
**Flow diagram of the progress through the phases of the randomized control trail**.

Participants were 206 Years 1–3 students in one large Primary school in the Perth Metropolitan area. Figure 1 illustrates progress through the phases of randomized control.

The double stream Primary School came from a very low socioeconomic (SES) status area in the Perth Metropolitan area and was placed in the 10th lowest decile of the H-Index which is used to rank SES in schools in Western Australia. The sample consisted of 85 boys and 107 girls (14 students did not give their gender), with approximately equal numbers of males and females across the conditions [intervention: boys (46.1%), girls (53.9 %); control: boys (42.2%), girls (57.8%)]. Participants were aged between 6 years 5 months, and 9 years 6 months.

To evaluate the pilot program for each year group, the 10 Year 1–3 classes were blocked into year groups. From within these three blocks, the classes were then randomly allocated to the intervention and usual care control conditions. The numbers of students in each year group in both intervention and control conditions are presented in Table 1. Students in the intervention group received the preventive program, whilst the control students received their regular health education lessons.

**Table 1:** **Number of students in each year and group**.

	**Year 1**	**Year 2**	**Year 3**
Intervention group	28	27	51
Control group	42	39	19

### Measures

#### Self-report measures

The *Children's Depression Inventory* (CDI; Kovacs, 1992) is a 27-item self-report measure that assesses cognitive, affective and behavioral symptoms of depression in children aged 7–17. Two-week test-retest reliability and internal consistency coefficients have been reported to be above 0.80 (Osman et al., 2010) with concurrent validity coefficients ranging from 0.66 to 0.81 (Kovacs, 1992). Item 9 (relating to suicidal ideation) was removed in accordance with the WA Department of Education's standards, resulting in a 26-item questionnaire. Other researchers who have similarly removed this item found internal consistency remained high (Cronbach's alpha = 0.89; Cole and Jordan, 1995; Cole et al., 2011).

The *Spence Children's Anxiety Scale* (SCAS; Spence, 1998; Essau et al., 2012b; Zhao et al., 2012) is a child self-report measure of symptoms relating to six subsets of anxiety disorders: social phobia, separation anxiety, obsessive-compulsive disorder, panic-agoraphobia, generalized anxiety and fears of physical injury in children aged 8 and above. The SCAS shows high internal consistency for the total scale as well as for each subscale, with coefficient alphas ranging from 0.60 to 0.92 (Spence, 1998). Test-retest reliability is moderate (0.63 after 3 months, and 0.60 after 6 months; (Spence, 1998)). Convergent validity with the Revised Children's Manifest Anxiety Scale (RCMAS) is acceptable (*r* = 0.71).

The *Assessment of Children's Emotional Skills* (ACES, Schultz et al., 2004) was used to assess children's emotional knowledge. The ACES is designed to measure emotion attribution accuracy in children aged 6 and older, and consists of three subscales concerning social behaviors, social situations, and facial expressions. Only the social behaviors and situations subscales were used in this study. Each subscale contains 15 items, in which children are asked to label the protagonist's feelings as “happy, sad, mad, scared, or no feeling.” Cronbach's alpha for the all the items across the three sections—social behaviors, situations, and facial expressions—is moderate (α = 0.68).

#### Parent measures

The Parent version of *Spence Children's Anxiety Scale* (SCAS-P; Spence, 1998) is designed to evaluate parents' perceptions of their child's level of anxiety symptoms. Satisfactory to excellent reliability and validity coefficients have been reported for all subscales, for both child and parent versions (Nauta et al., 2003). The SCAS-P was found to discriminate well between anxious and community children, and between the different anxiety disorders. Satisfactory to high internal consistency was found for the total scale (Cronbach's alpha = 0.89), and the six subscales (ranging from 0.55 to 0.82). Child reports were found to correlate moderately well with parent reports for internalizing symptoms (*r* = 0.66). The correlation between SCAS-P and CBCL-internalizing (*r* = 0.27) is significantly higher than the correlation between SCAS-P and CBCL-externalizing (*r* = 0.12; *z* = 3.15, *p* < 0.001), providing evidence for both divergent and convergent validity.

The parent form of the *Strengths and Difficulties Questionnaire* (SDQ-P; McCrory and Layte, 2012) consists of 25 items assessing conduct problems, hyperactivity, emotional symptoms, peer problems, and prosocial behavior. Goodman (2001) has reported sound Cronbach alpha coefficients for the sub-scales (mean = 0.73). Numerous studies have found the SDQ-P to have good concurrent and discriminant validity in non-clinical samples (Goodman, 2001; Muris et al., 2003; McCrory and Layte, 2012). The present study used the SDQ-P total difficulties (the sum of the conduct problems, hyperactivity, emotional symptoms, and peer problems scales) and SDQ-P prosocial scale.

#### Teacher measures

A 3-item scale teacher-rated Social Competence (SC-T) scale was constructed based on three pertinent domains of social competence, which were also the three aspects targeted by the program: social skills (e.g., verbal and nonverbal skills such as listening and turn taking), empathy, and social problem-solving. Teachers rated each child on a 5-point Likert-type scale (significantly below average to significantly above average). Test-retest reliability for the control group was high (*r* = 0.82). High internal consistency was also noted (Cronbach's alpha = 0.92 at pre-test and 0.95 at post-test). The SC-T was found to be significantly correlated with the prosocial scale of the SDQ-P (*r* = 0.25, *p* < 0.05).

### Feeling and friends program: manual production and program content

A literature review and Delphi analysis isolated the key components that should be targeted in the manual. The manual was written by the researcher and an educational specialist, who was an experienced Year 1–3 teacher. Contributions and editing were also provided by three experienced clinical psychologists/lecturers, two of whom developed the existing Aussie Optimism Programs. The Feelings and Friends program comprised 10 modules and was designed to support the Western Australian Health and Physical Education Curriculum, and to be implemented by class teachers to whole classes. The Feelings and Friends program was postulated to have its effect by targeting salient risk and protective factors for internalizing difficulties. Specifically, the program was designed to enhance social and emotional competence, in combination with equipping children with behavioral coping skills. Topics and brief content information of each module are detailed in Table 2.

**Table 2:** **Summary of feelings and friends modules**.

**Module**	**Topic**	**Aim of Module**
1	My feelings	For students to increase their emotional knowledge via normalizing emotions, and identifying a range of feelings
2	Body clues & first aid for feelings	For students to appreciate physiological manifestations of various emotions
		For students learn emotion regulation skills, be introduced to the idea that there are strategies that can be used to cope with unpleasant feelings, and learn to self soothe
3	First aid for being scared and worried	For students to learn and practice strategies to deal with anxiety (e.g., relaxation, confronting fear, pleasant activity scheduling)
4	First aid for anger	For students to learn and practice strategies to deal with fear (e.g., self soothe, STOP!)
5	Other people's feelings	For students to be aware of other people's feelings (e.g., by listening and looking for external body clues)
6	Caring about people's feelings	To foster empathy, and encourage respect and concern for other people's emotions
		For students to learn that different people may have different feelings in the same situation
7	Friendly habits	To reinforce prosocial behavior as well as identify and discourage anti-social behavior, particularly by fostering perspective taking and exercising empathy
8	Solving problems	For students to learn the process of problem-solving
9	Mad, sad & glad solutions	For student to be able to think about consequences of solutions in terms of their costs and benefits to the self and others
10	Quiz time!	Review knowledge, understandings, and skills developed throughout the program

### Procedure

This pilot trial of the Aussie Optimism Feeling and Friends program was carried out in conjunction with the larger scale Aussie Optimism whole school intervention. The volunteer school was in the 10th lowest decile of the H-Index, a measure of socio-economic status according to the Western Australian Government Department of Education and Training (McMillan and Western, 2000). A 2-stage consent process was employed, whereby active consent was sought from all parents of Years 1–3 children, followed by a passive consent procedure. Ninety-five percent of parents actively or passively consented to their child's participation. Initial data were collected at the start of Term 3 (Time 1), approximately a week prior to the start of the intervention.

The CDI and SCAS (given their recommended age range) were used with the Year 3 children only because of the poor reliability and validity noted on self-report measures with very young children (e.g., Myers and Winters, 2002; Chrisman et al., 2006). A list of questionnaires administered to children of the different age groups is presented in Table 3.

**Table 3:** **Questionnaires administered to each year group**.

**SELF REPORT MEASURES**	
CDI, SCAS	Year 3 only
ACES	Year 1–3
**PARENT REPORT MEASURES**	
SCAS-P, SDQ-P total difficulties, SDQ-P prosocial	Year 1–3
**TEACHER REPORT MEASURE**	
SC-T	Year 1–3

Students were provided with brief information about the project, and verbal assent to participate was sought. Standardized instruction and individual questions were read aloud to small groups or a class, by trained personnel who facilitated the administration and collection of questionnaires. Students were screened for clinical levels of depressive and anxious symptomology and parents of children who had elevated scores were confidentially contacted via the school. Relevant information and referral options were discussed with parents of children at risk. This process was repeated upon completion of the program in Term 4 (Time 2).

Training was provided to the teachers who agreed to be part of the study, and included detailing the rationale for promoting mental health within the school curriculum, teaching the various components in the program via direct instruction and role-playing, as well as supervised practice with the materials. Training was provided by the researcher and two registered clinical psychologists.

#### Process evaluation and social validity

To ensure the integrity of the program, facilitators of the program were asked to self-monitor their implementation of the program by completing a checklist and rating of the content covered in each module. Independent integrity measures were obtained by random observation of two of the teachers administering two different modules. This allowed for the comparison of teacher and expert ratings of program implementation, which provided a measure of implementation fidelity. Social validity aspects of the Years 1–3 program were examined by asking the students to rate their enjoyment of the program and parents to rate the effectiveness and utility of the program. Small focus groups were also held with students and teachers regarding usefulness, enjoyment of the program, suggested improvements and implementation issues.

### Data analysis

The psychometric data (ACES, CDI, SCAS, SCAS-P, SDQ-P total difficulties, SDQ-P pro-social, SC-T) were analyzed with a multi-level mixed effects linear regression model (Bryk and Raudenbush, 1987) as implemented through SPSS's Generalized Linear Mixed Models (GLMM: SPSS Version 19) procedure. The regression model was “mixed” in the sense that it included both random effects (child, school year) and fixed effects (group: intervention, control; time: pre-test, post-test). The regression model was “multi-level” in the sense that time was nested within child, and child was nested within school year. In order to optimize the likelihood of convergence, a separate GLMM analysis was run for each outcome. In each analysis, GLMM assumed a normal probability distribution for the outcome and linked it to the fixed effects (group, time, Group × Time) with an identity function. If the outcome did not have a normal distribution, then the parameter estimates of the covariance matrix were computed with robust statistics.

A clinical cut-off of 17 on the CDI has been used to indicate students who are *at-risk* of depression (Kovacs, 1992); and a clinical cut-off of 42 on the SCAS has been used to indicate students who are *at-risk* of anxiety (Kovacs, 1992). These cut-offs were used to classify children into healthy and at-risk groups at both pre-test and post-test. This generated four binary outcomes at post-test (post-test incidence of depression risk [yes/no]; post-test recovery from depression risk [yes/no]; post-test incidence of anxiety risk [yes/no]; post-test recovery from anxiety risk [yes/no]). The post-test binary outcomes were, once again, analyzed with a multi-level mixed effects regression model as implemented through SPSS's GLMM procedure. This time, however, GLMM used a binomial probability distribution for the outcomes and linked them to a single fixed effect (group) with a logit function. Once again, in order to optimize the likelihood of convergence, a separate GLMM analysis was run for each of the four binary outcomes.

Unlike repeated measures ANOVA (or ANCOVA), the multi-level mixed effects regression model does not rely on participants providing data at every assessment point; GLMM uses all the data present at each assessment point thereby reducing the impact of subject attrition on statistical power. Moreover, GLMM is relatively robust to unequal group sizes.

## Results

There was no child attrition at post-test for either group; however, 18 intervention parents and 11 control parents dropped out at post-test yielding attrition rates of 29 and 21%, respectively. There were no differences between the intervention and control drop-outs in terms of their pre-test outcome measures. Descriptive statistics for intervention and control groups on all outcomes are reported in Table 4.

**Table 4:** **Pre-test and Post-test means (sds) for intervention and control groups**.

**Group and Measure**	***N***	**Pre-test**	**Post-test**
**INTERVENTION**			
ACES	101	15.44 (2.99)	17.34 (3.33)
CDI (Year 3 only)	51	11.41 (8.05)	10.15 (9.56)
SCAS (Year 3 only)	51	32.75 (19.21)	30.89 (19.60)
SCAS-P	62	15.94 (8.80)	13.20 (7.02)
SDQ-P total difficulties	60	10.71 (7.10)	10.08 (6.08)
SDQ-P prosocial	61	7.82 (1.71)	7.84 (1.90)
SC-T	65	9.15 (2.10)	9.14 (2.37)
**CONTROL**			
ACES	84	14.39 (3.24)	15.45 (3.34)
CDI (Year 3 only)	15	12.62 (11.17)	11.06 (9.34)
SCAS (Year 3 only)	15	35.66 (18.61)	29.05 (17.40)
SCAS-P	53	14.48 (7.39)	14.61 (8.01)
SDQ-P total difficulties	53	9.88 (6.51)	9.80 (6.22)
SDQ-P prosocial	54	7.93 (1.73)	8.09 (1.72)
SC-T	61	8.87 (1.73)	8.44 (1.78)

### Primary analyses: student-reported outcomes

#### ACES

The Group × Time interaction was non-significant [*F*_(1, 375)_ = 3.39, *p* = 0.067, partial eta-squared = 0.009]. The group main effect was also non-significant [*F*_(1, 375)_ = 3.04, *p* = 0.082, partial eta-squared = 0.008]. There was, however, a significant main effect of time [*F*_(1, 375)_ = 66.68, *p* < 0.001, partial eta-squared = 0.151] indicating a significant pre-post improvement in emotional skills for both groups.

#### CDI

The Group × Time interaction was non-significant [*F*_(1, 127)_ = 0.02, *p* = 0.886, partial eta-squared = 0.000], as were the main effects of group [*F*_(1, 127)_ = 0.52, *p* = 0.474, partial eta-squared = 0.004], and time [*F*_(1, 127)_ = 1.12, *p* = 0.292, partial eta-squared = 0.009].

#### SCAS

The Group × Time interaction was non-significant [*F*_(1, 129)_ = 0.41, *p* = 0.525, partial eta-squared = 0.003], as were the main effects of group [*F*_(1, 129)_ = 0.02, *p* = 0.898, partial eta-squared = 0.000], and time [*F*_(1, 129)_ = 1.22, *p* = 0.272, partial eta-squared = 0.009].

#### At-risk analysis

There was no significant difference between groups in the proportion of students who moved from pre-test healthy to post-test at-risk (depression: *p* = 0.719; anxiety: *p* = 0.425), and no significant difference between groups in the proportion of students who moved from pre-test at-risk to post-test healthy (depression: *p* = 0.997; anxiety: *p* = 0.530).

### Secondary analyses: parent-reported and teacher-reported outcomes

#### SCAS-P

The Group × Time interaction was significant [*F*_(1, 197)_ = 4.43, *p* = 0.037, partial eta-squared = 0.022]. LSD (least significant difference) *post-hoc* contrasts indicated a significant pre-post decrease in anxiety symptoms for the intervention group (*p* = 0.009, partial eta-squared = 0.034), but no pre-post change for the control group (*p* = 0.708, partial eta-squared = 0.001).

#### SDQ-P total difficulties

The Group × Time interaction was non-significant [*F*_(1, 194)_ = 0.07, *p* = 0.793, partial eta-squared = 0.000], as were the main effects of group [*F*_(1, 194)_ = 0.85, *p* = 0.359, partial eta-squared = 0.005], and time [*F*_(1, 194)_ = 0.04, *p* = 0.837, partial eta-squared = 0.000].

#### SDQ-P social skills

The Group × Time interaction was non-significant [*F*_(1, 197)_ = 0.02, *p* = 0.893, partial eta-squared = 0.009], as were the main effects of group [*F*_(1, 197)_ = 0.12, *p* = 0.744, partial eta-squared = 0.001], and time [*F*_(1, 197)_ = 0.28, *p* = 0.595, partial eta-squared = 0.001].

#### SC-T

The Group × Time interaction was non-significant [*F*_(1, 215)_ = 0.12, *p* = 0.729, partial eta-squared = 0.001], as were the main effects of group [*F*_(1, 215)_ = 0.31, *p* = 0.577, partial eta-squared = 0.001], and time [*F*_(1, 215)_ = 0.00, *p* = 0.949, partial eta-squared = 0.000].

#### Gender effects

The Group × Time interaction reflects the intervention effect. A significant Gender × Group × Time interaction, therefore, would indicate that gender moderates the intervention effect. The three-way interaction was significant for SCAS-P [*F*_(1, 190)_ = 7.75, *p* = 0.006, partial eta-squared = 0.039]. *Post-hoc* LSD contrasts indicated a significant pre-post decrease in parent-reported anxiety for the intervention males (*p* = 0.004), in conjunction with a significant increase for the control males (*p* = 0.025). There was no pre-post change for the intervention females (*p* = 0.372) or the control females (*p* = 0.222). It appears that the previously reported pre-post decrease in parent-reported anxiety for the intervention group was carried by the males.

The Gender × Group × Time interaction was also significant for SC-T [*F*_(1, 193)_ = 8.20, *p* = 0.005, partial eta-squared = 0.041]. *Post-hoc* LSD contrasts indicated a significant pre-post increase in teacher-reported social competence for the intervention females (*p* = 0.041), in conjunction with a non-significant pre-post decrease for the control females (*p* = 0.112). There was a non-significant pre-post decrease for the intervention males (*p* = 0.079) and no change for the control males (*p* = 0.578).

### Program fidelity and integrity

In total, 100% of the modules were completed by two of the five intervention classes, 90% with one class, 80% with another, and 20% by the fifth class. As one of the classes completed only 20% of the modules, a multiple regression, controlling for pre-test levels, was conducted to determine whether there was a dosage effect that would warrant excluding this class from analysis. There was no evidence of a dosage effect for any of the measures (*p* > 0.05).

In some cases, not all of the components of a particular module were completed or the component was not completed as specified in the manual. Unfortunately, log books were not completed by two of the five facilitators. Therefore, it was unclear to what extent these classes completed all components. Of the three who completed the log books, at least 95% of each group session was completed either in full as presented or with modifications to suit student needs.

Complete independent integrity check data were only obtained for one of the facilitators. In this case, there was good agreement between the entries provided by the facilitator and observer.

### Process evaluation

Student enjoyment of the program was high, with 92% of children reporting that they enjoyed the program. Parents rated their children's enjoyment of the program highly (*M* = 3.9, *SD* = 1.1) on a scale of 1–5, with 1 indicating “not at all” and 5 indicating “very much.” Parents also rated a moderately high overall satisfaction of the program (*M* = 3.3, *SD* = 1.1) Parents reported moderate ratings of the effectiveness of the program (*M* = 2.9, *SD* = 1.2), and noticing positive changes in their children (*M* = 2.4, SD = 1.2). Few parents reported noticing negative changes in their children since participating in the program (*M* = 1.3, *SD* = 0.82). It appears though, that children did not talk to their parents about the program to a great extent (*M* = 2.0, *SD* = 1.0). As this may have impacted on parent's evaluation of the program, secondary analyses were conducted on students who were rated as having spoken to their parents to a fair degree (i.e., item rating ≥ 3). Parents from this sample rated their children's enjoyment more highly (*M* = 4.5, *SD* = 0.68) as well as overall usefulness of the program (*M* = 3.9, *SD* = 1.0).

Qualitative feedback was generally positive, with facilitators reporting implementation success across the Year 2 and 3 groups, and teachers of these year groups reporting that the experience was “worthwhile” and “has a lot of potential to help the children.” However, two teachers (of Year 1 classes) commented that the program was too difficult for their students and needed to be adapted and simplified.

## Discussion

The aim of the present study was to evaluate the short term efficacy of a universal school based prevention program that was piloted for the prevention of anxiety and depression in Years 1–3 children in an Australian primary school.

### Internalizing symptoms

#### Hypothesis 1a: universal prevention effects

With regards to internalizing symptoms, students in the prevention group did not show a greater decrease in depressive or anxiety symptoms compared with students in the control group. As such, the hypothesis that children in the prevention group would have significantly lower depression and anxiety scores as compared to children in the control group was not supported. This finding is in contrast with the findings of researchers targeting the prevention of internalizing disorders in older children, such as Barrett and Turner's (2001) universal prevention of anxiety program (FRIENDS for children), and Jaycox et al.'s (1994) selected study of depression program. In both studies, the intervention groups reported fewer internalizing symptoms at post-test than participants in the control condition. However, these results are compatible with Dadds et al.'s (1997) prevention of anxiety program (the QEIPAP which targeted younger children aged 7 years and above). In this study, Dadds and his colleagues did not find a significant difference between intervention and control groups in terms of anxiety symptoms immediately post intervention, with both groups showing improvements. However, this improvement in anxiety symptoms was only maintained in the intervention group at follow-up.

While no significant effects were found for child self-reports, parents of children in the prevention group reported a significantly greater decrease in their children's level of anxiety compared to parents of children in the control group. The issue of congruence between parent and child report has been problematic, with numerous researchers noting poor agreement between parent and child reports (e.g., Petot et al., 2011). Moreover, parent-child agreement was found to be lower with regards to internalizing symptoms (e.g., Rodriguez, 2011) as compared to observable behavior and externalizing symptoms (e.g., March et al., 1997 as cited in Nauta et al., 2003). Furthermore, with regards to concordance between parent and child interviews, poorer agreement was noted for younger as compared to older children (Klein, 1991; Petot et al., 2011). In determining which informant is more reliable in terms of diagnostic interviews, Klein (1991) concluded that “it is generally believed that the informant who provides the greater prevalence for a disorder is more likely to be accurate” (p. 196). Parents of children in the intervention condition, in contrast, reported significantly less anxiety issues in their children than those parents of children in the control condition and the effect size for this was in the small to moderate range. This finding partially supports Hypothesis 1a. It is unlikely that the significant findings observed in the parental reports were due to reactivity effects (i.e., parents in the prevention group responded in a biased manner because they were aware that their child was receiving the prevention program) as a similar improvement should have been evident on the other parent measure (SDQ) as well. Taken together, this may provide some preliminary support for the hypothesis that children in the prevention group exhibited less anxiety levels than children in the control group.

#### Hypothesis 1b: universal prevention effects

A second hypothesis was that children in the intervention group who scored in the normal range at pre-test would maintain a healthy status at post-test (Universal prevention effects). With regards to self-reported levels of anxiety or depression, the study provided no evidence that a greater proportion of children in the intervention group who score in the normal range at pre-test maintained their pre level scores.

#### Hypothesis 2: selected or indicated prevention effects

A third aim was to examine the prevention effects of the intervention on “at risk” children (Selected or Indicated prevention effects). It was predicted that a greater proportion of intervention children who exhibited symptoms of internalizing difficulties at pre-test would exhibit a decline in internalizing difficulties at post-test. There was no evidence of this found to support this hypothesis. This is not consistent with results found with the PENN program (e.g., Gillham et al., 2007) as well as the Aussie Optimism Program (e.g., Roberts et al., 2004). However, it is important to note that no follow up of these results have been carried out and effects may have been found at later time periods.

### Risk and protective factors: social and emotional competence

Children in the prevention group did not show a greater improvement in their emotional competence than children in the control group, which did not support the hypothesis that children in the prevention group would have more well-developed emotional knowledge at post-test than children in the control group, although again no follow-up was carried out where effects may have been found.

Teachers of students in the prevention group did not rate any greater improvement in their students' social competence than teachers of students in the control group. This is in contrast to findings by Kam et al. (2004). In this study, students who received PATHS displayed improvements in emotional competence (e.g., greater emotional understanding and recognition) but there was no significant differences in the intervention group in terms of teacher reported social competence. The authors postulated that this may have due to the program's stronger and early emphasis on emotional competence, and postulated that the program did not sufficiently focus on the acquisition of social competencies (Kam et al., 2004). Likewise, the current program initially focused on emotional competence (which is a natural prerequisite for social competence), and areas of social skills and social problem-solving were emphasized later on in the program. It is plausible that social competency skills and emotional skills may take time to be integrated, and may be more readily measurable at follow up testing. Another possible explanation for this lack of significant difference between groups in terms of gains in social competence may in part be attributed to the suspect validity of the social competence instrument employed in this study. This teacher measure of social competence may not be sufficiently sensitive to change.

Taken together, these results provide some support for the programs' potential preventive effect. The program appears to either directly or indirectly to ameliorate anxiety symptoms. It is hypothesized that the reduction of anxiety symptoms in the prevention group could come through direct teaching and application of behavioral strategies (e.g., using relaxation). Whether there are indirect pathways through improving emotional competence (e.g., general improvement in emotional regulation) or social competence remains to be evaluated via longer term follow-up and improved measures of social competence.

### Explanations for lack of significant findings

Perhaps one of the most obvious explanations for the lack of significant findings is the lack of program fidelity achieved by two of five teachers in the intervention group. The fact that the program was not completed or adhered to may have reduced the full potential of effects that might have been found. The lack of effect could be due to a latency effect, whereby a period of time is required for elements of the program (such as the strategies and concepts) to be adopted and integrated for effective use (Quayle et al., 2001). This latency effect has been found in several preventive studies such as Dadds et al.'s (1997) selective study, as well as in Quayle et al.'s (2001) and Roberts et al.'s (2003) universal studies. In these studies the critical effects were only apparent at 6 month follow-up. As such, it is plausible that the program's preventive effects could be more readily observable at a later time, when use of behavioral strategies and concepts become more practiced and generalized.

Another potential explanation for the lack of significant effects could be the relative brevity of the intervention and dilution of effects for anxiety and depression. It is feasible that the universal program spanning 10 sessions in total (the last being a review) is insufficient to bring about significant and lasting acquisition of protective skills.

Finally, the absence of significant effects for universal programs are not wholly unexpected, and are consistent with several research findings such as those from the meta-analysis conducted by Merry et al. (2003) and from Sheffield et al. (2006) evaluation of universal, indicated, and combined approaches.

Universal prevention programs generally require large sample sizes in order to capture clinically significant population effects. Cujipers (2003) estimated that 30, 211 participants across experimental and control conditions are required to capture a 15% decrease in the incidence of major depression, one of the common mental health disorders.

### Global social validity of the program

A further aim of the study was to ascertain the level of social validity and consumer satisfaction of the program so as to ensure the long-term viability of program. The vast majority of students reported that they enjoyed the program, with older students showing particularly good retention and being able to identify how the program was beneficial in their lives. Parents commented on the merits of the program, with some parents noting a number of positive changes in their children. Teacher feedback regarding the efficacy and usefulness of the program varied. Overall, the Years 2 and 3 teachers positively endorsed the program. However, the 2 Year One teachers who did not complete the program rated the program less favorably, stating the developmental inappropriateness of the program to their younger children being a major limitation. On the whole, child, parent and teacher feedback converge on the overall merits of such a prevention program, with the majority of teachers and students describing the experience as worthwhile and enjoyable.

## Limitations of the study and directions for future research

Several methodological limitations pertaining to the actual program and its implementation must be noted. One of the most important being the lack of program implementation fidelity for two of the five intervention classes. The program was run concurrently with at least one other universal program which meant that the demand on the teachers' resources was high. Thus, it is recommended that future programs be conducted in schools who are not simultaneously running other programs.

Another potential limitation was that the program appeared too complex for Year 1 and 2 students, many of whom are only just beginning to learn to read and write. The complexity of the program may have reduced the likelihood of gaining the full benefit for year 1 and 2 students so it is recommended that a separate developmentally appropriate with less reading and writing be developed for this age groups and the current version of the program maintained for Year 3 students.

There are several limitations pertaining to assessment issues. Firstly, self-report measures of internalizing symptoms were only available for the Year 3 children. This may have diluted the intervention effects that may have been found if self-report measures of internalizing difficulties were available for all year levels. In addition, as a result of random assignment, the reliance on only Year 3's resulted in a small control group, and the disproportionate sizes of both groups, may well have resulted in a corresponding loss of power. The decision not to use the self-report questionnaires also resulted in a reliance on parent measures of internalizing symptoms, where the response rate was not ideal. Secondly, the lack of program effects in terms of gains in social competence may in part be attributed to the validity of teacher ratings on the social competence measure.

Based on feedback regarding the developmental appropriateness and length of each session, future revisions of the Feelings and Friends program should target the length of the program by using more, but briefer modules. The program also needs to be simplified for the younger age group (incorporating Years 1 and 2). In addition, in light of the importance of soliciting family support and active familial engagement in the prevention process, it would be helpful to incorporate a parent component.

## Conclusions

There was some evidence suggesting that the program was able to prevent anxiety in children. However, longer term follow up is required in further trials to investigate whether the program has longer term effects on anxiety, depression, or social and emotional competence. Although this study suffered from some implementation and assessment limitations, these results, when taken in the context of previous research, provide grounds for optimism in the field of early prevention of internalizing disorders. This study highlights the importance and usefulness of targeting emotional wellbeing in young children. It also highlighted a number of program implementation issues that can guide future researchers on how to overcome some difficulties encountered in real-world applications of programs in schools. The findings of the present study provide evidence that with further modification, the Feelings and Friends program may be of benefit to the mental health of 6–8 year old children and it is clear that a larger trial of a developmentally modified program with follow up periods needs to be carried out.

## Author contributions

EP—Conducting the study and contribution in writing the manuscript draft. RR—Principal Investigator of the Healthway grant that funded the current project, contribution to introduction and discussion of the manuscript. RK—Methodology and data analysis. MN—Chief Investigator of the current project, revising the draft and contribution to the discussion section of the manuscript. MD—Revising the drafts and conceptualization of the project. NB—Contribution to introduction and discussion sections of the manuscript. SH—Revising drafts and contribution to the discussion section of the manuscript.

## Funding

This study was supported by the Western Australian Health Promotion Foundation (Healthway), through research grant (15148).

### Conflict of interest statement

The authors declare that the research was conducted in the absence of any commercial or financial relationships that could be construed as a potential conflict of interest.

## References

[B1] Anliak S. Sahin D. (2010). An observational study for evaluating the effects of interpersonal problem-solving skills training on behavioural dimensions. Early Child Dev. Care 180, 995–1003. 10.1080/03004430802670819

[B2] Barlow D. H. (2002). Anxiety and Its Disorders: The Nature and Treatment of Anxiety and Panic, 2nd Edn. New York, NY: Guildford Press.

[B3] Barrett P. Turner C. (2001). Prevention of anxiety symptoms in primary school children: preliminary results from a universal trial. Br. J. Clin. Psychol. 40, 399–410. 10.1348/014466501163887 11760616

[B4] Barrett P. M. Dadds M. R. Holland D. E. (1994). The Coping Koala: Prevention Manual. The University of Queensland, Queensland, Australia.

[B5] Bryk A. S. Raudenbush W. (1987). Application of hierarchical linear models to assessing change. Psychol. Bull. 101, 147–158. 10.1037/0033-2909.101.1.147

[B6] Bukowski W. M. Adams R. (2005). Peer relationships and psychopathology: markers, moderators, mediators, mechanisms, and meanings. J. Clin. Child Adolesc. Psychol. 34, 3–10. 10.1207/s15374424jccp3401_1 15677276

[B7] Calear A. L. Christensen H. (2010). Systematic review of school-based prevention and early intervention programs for depression. J. Adolesc. 33, 429–438. 10.1016/j.adolescence.2009.07.004 19647310

[B8] Chrisman A. Egger H. Compton S. N. Curry J. Goldston D. B. (2006). Assessment of childhood depression. Child Adolesc. Ment. Health 11, 111–116. 10.1111/j.1475-3588.2006.00395.x 32811084

[B9] Cole D. A. Jacquez F. M. Lagrange B. Pineda A. Q. Truss A. E. Weitlauf A. S.. (2011). A longitudinal study of cognitive risks for depressive symptoms in children and young adolescents. J. Early Adolesc. 31, 782–816. 10.1177/0272431610376248 25419034 PMC4238295

[B10] Cole D. A. Jordan A. E. (1995). Competence and memory: integrating psychosocial and cognitive correlates of child depression. Child Dev. 66, 459–473. 10.2307/1131590 7750377

[B11] Conner N. W. Fraser M. W. Soydan H. Sundell K. (2011). Preschool social–emotional skills training. Res. Soc. Work Pract. 21, 699–711. 10.1177/1049731511408115

[B12] Cujipers P. (2003). Examining the effects of prevention programs on the incidence of new cases of mental disorders: the lack of statistical power. Am. J. Psychiatry 160, 1385–1391. 10.1176/appi.ajp.160.8.1385 12900296

[B13] Dadds M. Seinen A. Roth J. Harnett P. (2000). Early Intervention for Anxiety Disorders in Children and Adolescents. Canberra, ACT: Commonwealth of Australia.

[B14] Dadds M. R. Holland D. E. Spence S. H. Laurens K. R. Mullins M. Barrett P. M. (1999). Early intervention and prevention of anxiety disorders in children: results at a 2-year follow-up. J. Consult. Clin. Psychol. 67, 145–150. 10.1037/0022-006X.67.1.145 10028219

[B15] Dadds M. R. Spence S. H. Holland D. E. Barrett P. M. Laurens K. R. (1997). Prevention and early intervention for anxiety disorders: a controlled trial. J. Consult. Clin. Psychol. 65, 627–635. 10.1037/0022-006X.65.4.627 9256564

[B16] Davis C. Martin G. Kosky R. O'Hanlon A. (2000). Early Intervention in the Mental Health of Young People: A Literature Review. Canberra, ACT: Commonwealth of Australia.

[B17] Dick-Niederhauser A. Silverman W. K. (2004). Prevention and early detection of emotional disorders, in Facilitating Pathways, eds Remschmidt H. Belfer M. L. Goodyear I. (Berlin: Springer), 272–286.

[B18] Donovan C. L. Spence S. H. (2000). Prevention of childhood anxiety disorders. Clin. Psychol. Rev. 20, 509–531. 10.1016/S0272-7358(99)00040-9 10832552

[B19] Durlak J. A. Weissberg R. P. Dymnicki A. B. Taylor R. D. Schellinger K. B. (2011). The impact of enhancing students' social and emotional learning: a meta-analysis of school-based universal interventions. Child Dev. 82, 405–432. 10.1111/j.1467-8624.2010.01564.x 21291449

[B20] Dymond S. Schlund M. W. Roche B. Whelan R. Richards J. Davies C. (2011). Inferred threat and safety: symbolic generalization of human avoidance learning. Behav. Res. Ther. 49, 614–621. 10.1016/j.brat.2011.06.007 21767825

[B21] Eisenberg S. A. Shen B. J. Schwarz E. R. Mallon S. (2012). Avoidant coping moderates the association between anxiety and patient-rated physical functioning in heart failure patients. J. Behav. Med. 35, 253–261. 10.1007/s10865-011-9358-0 21660588

[B22] Essau C. A. Conradt J. Sasagawa S. Ollendick T. H. (2012a). Prevention of anxiety symptoms in children: results from a universal school-based trial. Behav. Ther. 43, 450–464. 10.1016/j.beth.2011.08.003 22440079

[B23] Essau C. A. Olaya B. Pasha G. O'Callaghan J. Bray D. (2012b). The structure of anxiety symptoms among adolescents in Iran: a confirmatory factor analytic study of the Spence Children's Anxiety Scale. J. Anxiety Disord. 26, 871–878. 10.1016/j.janxdis.2012.08.001 23070031

[B24] Fine S. E. Izard C. E. Mostow A. J. Trentacosta C. J. Ackerman B. P. (2003). First grade emotion knowledge as a predictor of fifth grade self-reported internalising behaviours in children from economically disadvantaged families. Dev. Psychopathol. 15, 331–342. 10.1017/S095457940300018X 12931831

[B25] Fox J. K. Masia Warner C. Lerner A. B. Ludwig K. Ryan J. L. Colognori D.. (2012). Preventive intervention for anxious preschoolers and their parents: strengthening early emotional development. Child Psychiatry Hum. Dev. 43, 544–559. 10.1007/s10578-012-0283-4 22331442 PMC3759969

[B26] Gagnon S. G. Nagle R. J. (2004). Relationships between peer interactive play and social competence in at-risk preschool children. Psychol. Schools 41, 173–189. 10.1002/pits.10120

[B27] Gillham J. E. Reivich K. J. Freres D. R. Chaplin T. M. Shatté A. J. Samuels B.. (2007). School-based prevention of depressive symptoms: a randomized controlled study of the effectiveness and specificity of the Penn Resiliency Program. J. Consult. Clin. Psychol. 75, 9–19. 10.1037/0022-006X.75.1.9 17295559 PMC4469032

[B28] Glick G. C. Rose A. J. (2011). Prospective associations between friendship adjustment and social strategies. Dev. Psychol. 47, 1117–1132. 10.1037/a0023277 21443336 PMC3389512

[B29] Goodman R. (2001). Psychometric properties of the strengths and difficulties questionnaire. J. Am. Acad. Child Adolesc. Psychiatry 40, 1337–1345. 10.1097/00004583-200111000-00015 11699809

[B30] Goodwin N. P. Mrug S. Borch C. Cillessen A. H. (2012). Peer selection and socialization in adolescent depression: the role of school transitions. J. Youth Adolesc. 41, 320–332. 10.1007/s10964-011-9723-x 22009309 PMC3277679

[B31] Grav S. Hellzèn O. Romild U. Stordal E. (2012). Association between social support and depression in the general population: the HUNT study, a cross-sectional survey. J. Clin. Nurs. 21, 111–120. 10.1111/j.1365-2702.2011.03868.x 22017561

[B32] Greenberg M. T. Weissberg R. P. O'Brien M. U. Zins J. E. Fredericks L. Resnik H.. (2003). Enhancing school-based prevention and youth development through coordinated social, emotional, and academic learning. Am. Psychol. 58, 466–474. 10.1037/0003-066X.58.6-7.466 12971193

[B33] Hansell N. K. Wright M. J. Medland S. E. Davenport T. A. Wray N. R. Martin N. G.. (2012). Genetic co-morbidity between neuroticism, anxiety/depression and somatic distress in a population sample of adolescent and young adult twins. Psychol. Med. 42, 1249–1260. 10.1017/S0033291711002431 22051348

[B34] Heaney J. L. Phillips A. C. Carroll D. (2010). Ageing, depression, anxiety, social support and the diurnal rhythm and awakening response of salivary cortisol. Int. J. Psychophysiol. 78, 201–208. 10.1016/j.ijpsycho.2010.07.009 20688111

[B35] Hessler D. M. Katz L. F. (2010). Brief report: associations between emotional competence and adolescent risky behavior. J. Adolesc. 33, 241–246. 10.1016/j.adolescence.2009.04.007 19481247 PMC2822004

[B36] Hirshfeld-Becker D. R. Masek B. Henin A. Blakely L. R. Pollock-Wurman R. A. McQuade J.. (2010). Cognitive Behavioral Therapy for 4 to 7-year-old children with anxiety disorders. J. Consult. Clin. Psychol. 78, 498–510. 10.1037/a0019055 20658807

[B37] Jakobsen I. Horwood L. Fergusson D. (2012). Childhood anxiety/withdrawal, adolescent parent–child attachment and later risk of depression and anxiety disorder. J. Child Fam. Stud. 21, 303–310. 10.1007/s10826-011-9476-x

[B38] Jaycox L. H. Reivich K. J. Gillham J. Seligman M. E. (1994). Prevention of depressive symptoms in school children. Behav. Res. Ther. 32, 801–816. 10.1016/0005-7967(94)90160-0 7993324

[B39] Johnstone J. Rooney R. M. Hassen S. Kane R. T. (2014). Prevention of depression and anxiety symptoms in adolescents: 42 and 54 months follow-up of the Aussie Optimism Program- Positive Thinking Skills. Front. Psychol. 5:364. 10.3389/fpsyg.2014.00364 24904446 PMC4036073

[B40] Kam C. Greenberg M. T. Kusche C. A. (2004). Sustained effects of the PATH curriculum on the social and psychological: adjustment of children in special education. J. Emot. Behav. Disord. 12, 66–78. 10.1177/10634266040120020101

[B41] Klein D. N. Leon A. C. Li C. D'Zurilla T. J. Black S. R. Vivian D.. (2011). Social problem solving and depressive symptoms over time. J. Consult. Clin. Psychol. 79, 342–352. 10.1037/a0023208 21500885 PMC3109172

[B42] Klein R. G. (1991). Parent-child agreement in clinical assessment of anxiety and other psychopathology: a review. J. Anxiety Disord. 5, 187–198. 10.1016/0887-6185(91)90028-R

[B43] Kochel K. P. Miller C. F. Updegraff K. A. Ladd G. W. Kochenderfer-Ladd B. (2012). Associations between fifth graders' gender atypical problem behavior and peer relationships: a short-term longitudinal study. J. Youth Adolesc. 41, 1022–1034. 10.1007/s10964-011-9733-8 22113585

[B44] Kösters M. P. Chinapaw M. J. Zwaanswijk M. van der Wal M. F. Utens E. M. Koot H. M. (2012). Study design of ‘FRIENDS for Life’: process and effect evaluation of an indicated school-based prevention programme for childhood anxiety and depression. BMC Public Health 12:86. 10.1186/1471-2458-12-86 22284741 PMC3292977

[B45] Kovacs M. (1992). Children's Depression Inventory. Ney Work, NY: Multi-Health Systems.

[B46] Linaries L. O. Rosbruch N. Stern M. B. Edwards M. E. Walker G. Abikoff H. B. (2005). Developing cognitive-social-emotional competencies to enhance academic learning. Psychol. Schools 42, 405–417. 10.1002/pits.20066

[B47] Lowry-Webster H. Barrett P. Dadds M. (2001). A universal prevention trial for anxiety and depressive symptomatology in childhood: preliminary data from an Australian study. Behav. Change 18, 36–50. 10.1375/bech.18.1.36

[B48] March J. S. Parker J. D. Sullivan K. Stallings P. Conners C. K. (1997). The multidimensional anxiety scale for children (MASC): factor structure, reliability, and validity. J. Am. Acad. Child Adolesc. Psychiatry 36, 554–565. 10.1097/00004583-199704000-00019 9100431

[B49] Masten A. S. (2005). Peer relationships and psychopathology in developmental perspective: reflections on progress and promise. J. Clin. Child Adolesc. Psychol. 34, 87–92. 10.1207/s15374424jccp3401_8 15677283

[B50] McCrory C. Layte R. (2012). Testing competing models of the Strengths and Difficulties Questionnaire's (SDQ's) factor structure for the parent-informant instrument. Person. Indiv. Diff. 52, 882–887. 10.1016/2012.02.011

[B51] McLoone J. Hudson J. L. Rapee R. M. (2006). Treating anxiety disorders in a school setting. Educ. Treat. Child. 29, 219–232.

[B52] McMillan J. Western J. (2000). Measurement of the socio-economic status of Australian higher education students. Higher Educ. 39, 223–248. 10.1023/A:1003943824357

[B53] Muris P. Meester C. van den Berg, C. (2003). The Strengths and Difficulties (SDQ) further evidence for its reliability and validity in a community sample of Dutch children and adolescents. Eur. Child Adolesc. Psychiatry 12, 1–8. 10.1007/s00787-003-0298-2 12601558

[B54] Merry S. McDowell H. Hetrick S. Bir J. Muller N. (2003). Psychological and/or educational interventions for the prevention of depression in children and adolescents (Review). Cochrane Database Syst Revi 2, 1–16. 10.1002/14651858.CD003380.pub2 14974014

[B55] Miller A. L. Fine S. E. Gouley K. K. Seifer R. Dickstein S. Shields A. (2006). Showing and telling about emotions: interrelations between facets of emotional competence and associations with classroom adjustment in Head Start preschoolers. Cogn. Emot. 20, 1170–1192. 10.1080/02699930500405691

[B56] Monshouwer K. Smit F. Ruiter M. Ormel H. Verhulst F. Vollebergh W.. (2012). Identifying target groups for the prevention of depression in early adolescence: the TRAILS study. J. Affect. Disord. 138, 287–294. 10.1016/j.jad.2012.01.026 22341484

[B57] Myers K. Winters N. C. (2002). Ten-year review of rating scales. II: scales for internalizing disorders. J. Am. Acad. Child Adolesc. Psychiatry 41, 634–659. 10.1097/00004583-200206000-00004 12049439

[B58] Myles-Pallister J. D. Hassen S. Rooney R. M. Kane R. (2014). The efficacy of the enhanced Aussie Optimism Positive Thinking Skills Program in improving social and emotional learning in middle childhood. Front. Psychol. 5:909. 10.3389/fpsyg.2014.00909 25177310 PMC4133646

[B59] Nauta M. H. Scholing A. Rapee R. M. Abbott M. Spence S. H. Waters A. (2003). A parent-report measure of children's anxiety: psychometric properties and comparison with child-report in a clinic and normal sample. Behav. Res. Ther. 42, 813–829. 10.1016/S0005-7967(03)00200-6 15149901

[B60] Oades-Sese G. V. Esquivel G. B. Kaliski P. K. Maniatis L. (2011). A longitudinal study of the social and academic competence of economically disadvantaged bilingual preschool children. Dev. Psychol. 47, 747–764. 10.1037/a0021380 21219064

[B61] Osman A. Gutierrez P. M. Bagge C. L. Fang Q. Emmerich A. (2010). Reynolds adolescent depression scale- second edition: a reliable and useful instrument. J. Clin. Psychol. 66, 1324–1345. 10.1002/jclp.20727 20715023

[B62] Petot D. Rescorla L. Petot J. M. (2011). Agreement between parent- and self-reports of Algerian adolescents' behavioral and emotional problems. J. Adolesc. 34, 977–986. 10.1016/j.adolescence.2010.11.011 21163517

[B63] Pössel P. Adelson J. L. Hautzinger M. (2011). A randomized trial to evaluate the course of effects of a program to prevent adolescent depressive symptoms over 12 months. Behav. Res. Ther. 49, 838–851. 10.1016/j.brat.2011.09.010 22030296

[B64] Quayle D. Dziurawiec S. Roberts C. Kane R. Ebsworthy G. (2001). The effects of an optimism and life skills program on depression in pre-adolescents. Behav. Change 18, 194–203. 10.1375/bech.18.4.194

[B65] Roberts C. M. Bishop B. (2003). Depression, Childhood, in Encyclopaedia of Primary Prevention and Health Promotion, eds Guilotta T. P. Bloom M. (New York, NY: Kluwer Academic/Plenum Publishers), 395–403.

[B66] Roberts C. Kane R. Bishop B. Mathews H. Thomson H. (2004). The prevention of depression in rural school children: a follow-up study. Int. J. Ment. Health Promot. 6, 4–16. 10.1080/14623730.2004.9721934

[B67] Roberts C. Kane R. Thomson H. Bishop B. Hart B. (2003). The prevention of depression in rural school children: a randomised controlled trial. J. Consult. Clin. Psychol. 71, 622–628. 10.1037/0022-006X.71.3.622 12795585

[B68] Rodebaugh T. L. Holaway R. M. Heimberg R. G. (2004). The treatment of social anxiety disorder. Clin. Psychol. Rev. 24, 883–908. 10.1016/j.cpr.2004.07.007 15501560

[B69] Rodriguez C. (2011). Association between independent reports of maternal parenting stress and children's internalizing symptomatology. J. Child Fam. Stud. 20, 631–639. 10.1007/s10826-010-9438-8

[B70] Romeo R. Knapp M. Banerjee S. Morris J. Baldwin R. Tarrier N.. (2011). Treatment and prevention of depression after surgery for hip fracture in older people: cost-effectiveness analysis. J. Affect. Disord. 128, 211–219. 10.1016/j.jad.2010.07.026 20696480

[B71] Rooney R. Hassen S. Kane R. Roberts C. M. Nesa M. (2013a). Reducing depression in 9-10 year old children in low SES schools: a longitudinal universal randomized controlled trial. Behav. Res. Ther. 51, 845–854. 10.1016/j.brat.2013.09.005 24185214

[B72] Rooney R. Morrison D. Hassen S. Kane R. Roberts C. Mancini V. (2013b). Prevention of internalizing disorders in 9-10 year old children: efficacy of the Aussie Optimism Positive Thinking Skills Program at 30-month follow-up. Front. Psychol. 4:988. 10.3389/fpsyg.2013.00988 24421776 PMC3872743

[B73] Rooney R. Roberts C. Kane R. Pike L. Winsor A. White J. (2006). The prevention of depression in 8-9 year-old children: a pilot study. Aust. J. Guid. Couns. 16, 76–90. 10.1375/ajgc.16.1.76

[B74] Saltali N. D. Deniz M. E. (2010). The effects of an emotional education program on the emotional skills of six-year-old children attending preschool. Educ. Sci. 10, 2123–2140.

[B75] Schultz D. Izard C. E. Bear G. (2004). Children's emotion processing: relations to emotionality and aggression. Dev. Psychopathol. 16, 371–387. 10.1017/S0954579404044566 15487601

[B76] Sheffield J. K. Spence S. H. Rapee R. M. Kowalenko N. Wignall A. Davis A. (2006). Evaluation of a universal, indicated, and combined cognitive-behavioural approaches to the prevention of depression among adolescents. J. Consult. Clin. Psychol. 74, 66–79. 10.1037/0022-006X.74.1.66 16551144

[B77] Shure M. B. (1997). Interpersonal Cognitive Problem Solving: Primary Prevention of Early High-Risk Behaviors in the Preschool and Primary Years. Thousand Oaks, CA: Sage Publications, Inc.

[B78] Spence S. H. (1998). A measure of anxiety symptoms among children. Behav. Res. Ther. 36, 545–566. 10.1016/S0005-7967(98)00034-5 9648330

[B79] Spence S. H. (2003). Social skills training with children and young people: theory, evidence and practice. Child Adolesc. Ment. Health 8, 84–96. 10.1111/1475-3588.00051 32797550

[B80] Tomb M. Hunter L. (2004). Prevention of anxiety in children and adolescents in a school setting: the role of school-based practitioners. Child. Schools 26, 87–101. 10.1093/cs/26.2.87

[B81] Tonmyr L. Williams G. Hovdestad W. E. Draca J. (2011). Anxiety and/or depression in 10–15-year-olds investigated by child welfare in Canada. J. Adolesc. Health 48, 493–498. 10.1016/j.jadohealth.2010.08.009 21501809

[B82] Trudeau L. Spoth R. Randall G. K. Mason W. A. Shin C. (2012). Internalizing symptoms: effects of a preventive intervention on developmental pathways from early adolescence to young adulthood. J. Youth Adolesc. 41, 788–801. 10.1007/s10964-011-9735-6 22160441 PMC3771682

[B83] Tsai A. C. Bangsberg D. R. Frongillo E. A. Hunt P. W. Muzoora C. Martin J. N.. (2012). Food insecurity, depression and the modifying role of social support among people living with HIV/AIDS in rural Uganda. Soc. Sci. Med. 74, 2012–2019. 10.1016/j.socscimed.2012.02.033 22513248 PMC3348339

[B84] Watson D. Gamez W. Simms L. J. (2005). Basic dimensions of temperament and their relation to anxiety and depression: a symptom-based perspective. J. Res. Pers. 39, 46–66. 10.1016/j.jrp.2004.09.006

[B85] Webster-Stratton C. Reid M. J. (2004). Strengthening social and emotional competence in young children – The foundation for early school readiness and success. Infants Young Child. 17, 96–113. 10.1097/00001163-200404000-00002

[B86] Wright M. Banerjee R. Hoek W. Rieffe C. Novin S. (2010). Depression and social anxiety in children: differential links with coping strategies. J. Abnorm. Child Psychol. 38, 405–419 10.1007/s10802-009-9375-4 20012184

[B87] Zhao J. Xing X. Wang M. (2012). Psychometric properties of the Spence Children's Anxiety Scale (SCAS) in Mainland Chinese children and adolescents. J. Anxiety Disord. 26, 728–736. 10.1016/j.janxdis.2012.05.006 22858899

